# Linkage analysis of complex diseases using microsatellites and single-nucleotide polymorphisms: application to alcoholism

**DOI:** 10.1186/1471-2156-6-S1-S10

**Published:** 2005-12-30

**Authors:** Jérémie Nsengimana, Hélène Renard, David Goldgar

**Affiliations:** 1 Genetic Epidemiology Group, International Agency for Research on Cancer, World Health Organization, 150 Cours Albert Thomas, 69008 Lyon, France; 2 Saint James's University Hospital, Genetic Epidemiology Division, Cancer Research UK Clinical Centre in Leeds, Cancer Genetics Building, Beckett Street, Leeds LS9 7TF, UK

## Abstract

The efficacy of linkage studies using microsatellites and single-nucleotide polymorphisms (SNPs) was evaluated. Analyzed data were supplied by the Collaborative Study on the Genetics of Alcoholism (COGA). Alcoholism was analyzed together with a simulated trait caused by a gene of known position, through a nonparametric linkage test (NPL). For the alcoholism trait, four densities of SNPs (1 SNP per 0.2 cM, 0.5 cM, 1 cM and 2 cM) showed higher peaks of NPL *z *scores and smaller significant *p*-values than the usual 10-cM density of microsatellites. However, the two highest densities of SNPs had unstable z score signals, and therefore were difficult to interpret. Analyzing a simulated trait with the same markers in the same pedigrees, we confirmed the higher power of all four densities of SNPs compared to the 10-cM microsatellites panel, although the existence of other confounding peaks was confirmed for maps that are denser than 1 SNP/cM. We further showed that estimating the gene position using SNPs is far less biased than using the usual panel of microsatellites (biases of 0–2 cM for SNPs vs. 8.9 cM for microsatellites). We conclude that using dense maps of SNPs in linkage analysis is more powerful and less biased than using the 10-cM maps of microsatellites. However, linkage signals can be unstable and difficult to interpret when several SNPs are genotyped per centimorgan. The power and accuracy of 1 SNP/cM or 1 SNP/2 cM may be sufficient in a genome-wide linkage scan while denser maps may be most useful in fine-gene mapping studies exploiting linkage disequilibrium.

## Background

A number of genome-wide linkage studies reported chromosome regions that may harbor genes for alcoholism. Most of these studies [1,2] used data of the Collaborative Study on the Genetics of Alcoholism (COGA), consisting of microsatellite markers genotyped in more than 100 multigenerational families. One of the mapping approaches used was the nonparametric test (NPL) of linkage [3]. Considering the binary trait ALDX1 (DSM-III-R and the Feighner criteria for alcoholism), regions of significant or suggestive NPL *z *scores were found on chromosomes 1, 6, 7, and 15 [2]. For the purpose of the Genetic Analysis Workshop 14 (GAW14), COGA supplied microsatellites and single nucleotide polymorphisms (SNP) genotyped in 142 families. The aim of our study was to compare the efficiency of NPL analysis using microsatellites and SNPs, applied to the alcohol dependence trait ALDX1 and to an artificial simulated trait caused by a gene of known position.

## Methods

### Subjects

The COGA definition of "pure affecteds" and "pure unaffecteds" was considered, i.e., individuals with only a few symptoms were considered as unknown phenotype. Because the COGA dataset is multiethnic, we analyzed only the "White non-Hispanic" group, following Sheffield et al. [4] and Windemuth et al. [5], in order to avoid the heterogeneity that may be caused by the ethnic origin. "White non-Hispanic" was the most represented group in the dataset, with 102 of the 142 families. The total number of individuals in these families is 1,074. The affection status on ALDX1 was available in 616 individuals (57.4%), of which 444 were affected (72.1%).

### Genotypes

We focused on chromosome 1 because previous studies pointed toward regions on this chromosome as potentially harboring genes for alcoholism [2]. Two types of data were analyzed: 27 microsatellites covering 279.2 cM (Kosambi map) and 250 SNPs typed on a segment of this chromosome between position 99.512 cM and position 154.672 cM (Affymetrix SNP 251 to 500). For SNP data, four different densities were analyzed: a mean marker spacing of 2 cM, 1 cM, 0.5 cM, and 0.2 cM. The mean spacing of 0.2 cM corresponds to the whole set of Affymetrix SNPs genotyped on the considered region (250 SNPs along ~55 cM). However, SNPs were not uniformly distributed along the chromosome: 20% of SNPs shared the same position as some others (in recombination units) while the largest distance between two consecutive SNPs was 2.55 cM. Mean spacings of 2 cM, 1 cM and 0.5 cM were achieved by excluding one of any two SNPs that were separated by a distance smaller than a fixed threshold. For example to obtain a mean distance of 1 cM, we defined 0.6 cM as the minimum distance between any two adjacent SNPs. The SNP that was excluded was selected randomly.

### Statistical analyses

The software MERLIN (Multipoint Engine for Rapid Likelihood Inference, see [6]) was used to perform a NPL test based on allele sharing between all affected family members. This program checks the possible errors in data and excludes erroneous genotypes from analyses.

### Simulation

To assess the accuracy and precision of NPL analysis using microsatellites versus SNPs, we simulated a susceptibility locus for a binary trait at a known position by choosing one SNP that lies at position 118.09 cM (Kosambi map) on chromosome 1 (the SNP tsc1596419). Only the trait was simulated; the real pedigree and all individual genotypes from COGA (Affymetrix SNPs) were kept. The minor allele of the trait locus (frequency of 0.04 in the White population) was considered as the susceptibility allele. A dominant model with complete penetrance and no phenocopies was simulated, thus the trait prevalence was 8%. We then localized this locus with the same mapping approach as with the real data. Though the genetic model simulated was simple, we believe that it can give a clear indication on the mapping accuracy using SNPs and microsatellites. Because we did not know the true location of an alcoholism gene, we could not address this question using the COGA data. The idea is that if one approach behaves poorly in a simple Mendelian disease, it should not be more powerful in complex diseases.

## Results

### The alcohol dependence trait ALDX1

**Figure 1:**
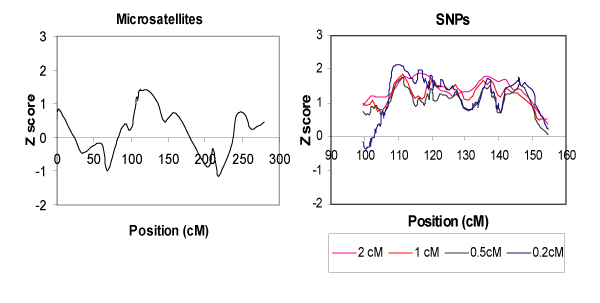
**The *z *scores of NPL analyses of microsatellites and SNP data. **The *p*-values corresponding to the highest peaks are 0.08 for microsatellites and 0.02–0.04 for SNPs.

The observed *z *scores of NPL with microsatellites and different densities of SNPs are plotted on Figure 1. The maximum score obtained with microsatellites is 1.40 (*p *= 0.08) in the region 111–122 cM. The *z *scores observed with SNPs are higher than those of microsatellites: the highest score varies between 1.76 and 2.14 (*p *= 0.02 to 0.04, see Figure 1 and Table 1). For all four densities of SNPs, the highest peak of *z *score is between positions 108 cM and 112 cM, except the density of 2 cM, which has the highest peak at 114–118 cM. One to six additional peaks of smaller height are observed depending on the marker density; some of them had nominally significant *p*-values (Table 1). However, some of these peaks are so close to each other that their actual delineation should be interpreted carefully (e.g., with density 0.2 cM).

### The simulated trait

**Figure 2:**
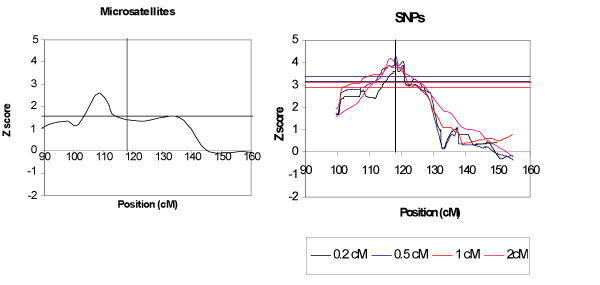
**Plots of *z *scores obtained with the simulated trait. **The vertical line indicates the actual position of the simulated gene and horizontal lines correspond to the threshold values given by one-*z*-score drop from the peak of the curves. The *p*-values corresponding to the highest peaks are 0.005 for microsatellites and 2 × 10^-5 ^to 7 × 10^-5 ^for different panels of SNPs.

Running the NPL test to map the simulated gene with microsatellites, the observed highest *z *score was 2.57 (*p *= 0.005) but its position was at 109.2 cM, while the true position of the simulated gene was 118.09 cM (Figure 2). There was therefore a downward bias of 8.89 cM. The one-*z*-score interval around the estimated position is from 103 cM to 115 cM and does not contain the actual simulated position (see Figure 2). With the four densities of SNPs, the peak of *z *score was 3.88 to 4.39 (Figure 2), with *p*-values of 2 × 10^-5 ^to 7 × 10^-5^. These peaks of *z *score were observed at the exact position of the simulated gene for the mean densities of 1 SNP/0.2 cM and 1 SNP/0.5 cM, while it was within an interval of 2 cM from this position for mean densities of 1 SNP/1 cM and 1 SNP/2 cM (Figure 2). The one-*z*-score interval contained the actually simulated position for all four SNP densities. However, for the two highest densities (0.2 cM and 0.5 cM), one additional peak was observed at position 121 cM (see Figure 2). This peak does not correspond to the simulated locus. It confirms the potential of very dense maps to give confusing results with the presently available methods of analysis.

## Discussion

Unlike studies of GAW11, which reported significant and suggestive NPL scores on chromosome 1 obtained with GENEHUNTER (GH) and GENEHUNTER-plus (GH+) [2], MERLIN failed to find a nominally significant gene for alcoholism on this chromosome using microsatellites data (Figure 1). Though MERLIN may be more conservative than GH and GH+, another possible cause of differences in these results is the genetic maps used. We observed that some of the microsatellites used in GAW11 were not genotyped for GAW14 while some new ones were added. Also, the length of the chromosome differed between studies [7]. Nevertheless, we noted that the region showing peaks of *z *scores in our study corresponds to one of the regions that showed a significant or a suggestive *z *score with GH and GH+ [2]. This is why we decided to concentrate our attention to this region when analyzing SNP data.

Four map resolutions of SNPs were evaluated, with mean spacing of 0.2 cM, 0.5 cM, 1 cM, and 2 cM between adjacent SNPs. We observed higher *z *scores and lower *p*-values with any of these densities of SNPs than with microsatellites (Figure 1). This can be explained by the extra information supplied by the new genotyped locations. However, as the map resolution increased, the number of peaks of the *z *score curve also increased (Figure 1 and Table 1), and it is not clear whether all of these peaks correspond to different putative genes. The density of 0.5 cM indicated only one significant peak, although the corresponding *z *score was one of the lowest (Table 1), while the densities of 1 cM and 2 cM show three and two peaks, respectively, with *p*-values smaller or equal to 0.05. We recommend caution when using very high densities in linkage analysis, due to the cost of such studies and to possible difficulties in drawing conclusions after the analysis.

**Figure 3:**
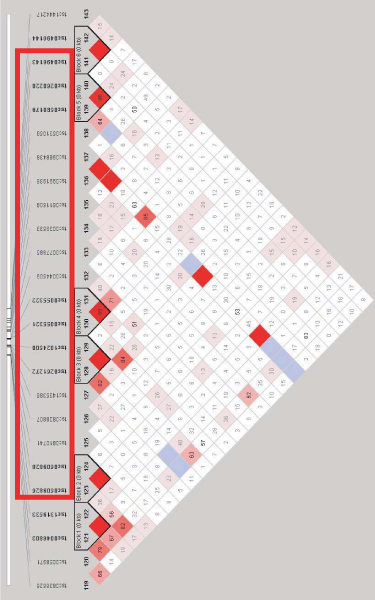
**Haplotype blocks with the density of 1 SNP/0.2 cM. **The red color indicates a high D' (>0.80) and a high corresponding LOD score, the blue color indicates a high D' but with a low corresponding LOD score. The pink and white colors indicate a low D' [8]. With this density, six haplotype blocks are found on a region of 4.378 cM, between 121.85 cM and 126.228 cM.

**Figure 4:**
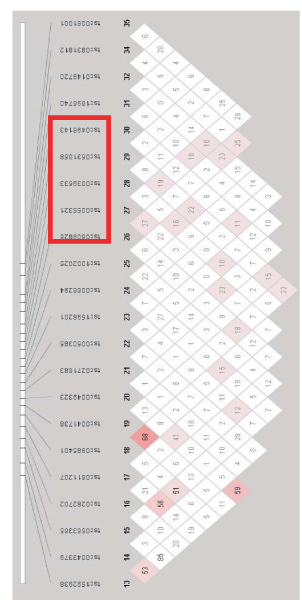
**Haplotype blocks with the density of 1 SNP/cM. **The colors signification is the same as on Figure 3. The region displayed is between position 110 cM and 130 cM, where the density 1 SNP/0.2 cM showed multiple linkage peaks (See Figure 1). Zero haplotype blocks were found on all the area using this density.

One possible source of confusion when the SNPs are very close to each other is linkage disequilibrium (LD). In fact, tests of linkage analysis assume the absence of LD and when it exists, it can affect the results of these tests. Figures 3 and 4 illustrate a haplotype block analysis with the program HAPLOVIEW [8] when average densities of 1 SNP/0.2 cM and 1 SNP/1 cM were considered, respectively. To define a haplotype block, we used the confidence interval approach of Gabriel et al. [9]. The red rectangle on these pictures highlights the same analyzed region. Up to six blocks can be observed for the density 1 SNP/0.2 cM (Figure 3), while none was found for the density of 1 SNP/1 cM (Figure 4). Blocks 3 and 4 on Figure 3 are located in the area of a linkage peak that was observed only with density 1 SNP/0.2 cM (between 123 and 124 cM, see Table 1 and Figure 1). Strong LD between very close SNPs may then explain the fluctuation of linkage signals. Overall, in the region comprised between 110 cM and 130 cM (the region of multiple NPL peaks, see Figure 1), 10 haplotype blocks were found using the density of 1 SNP/0.2 cM. Nonetheless, no blocks were found in some large regions of the chromosome even using the full COGA dataset, due to its relatively small resolution. Indeed, the average size of haplotype blocks in European populations was shown to be in the order of 22 kb [9], 10-fold smaller than the average resolution of the Affymetrix maps. Therefore, rather than using tag-SNPs, we used fixed, arbitrary densities in our analysis and we selected SNPs randomly when they shared the position. This approach corresponds to a homogeneous distribution of SNPs along the chromosome. Indeed, in practice, if one plans to perform a genome-wide scan without any knowledge of the location of recombinational hotspots and cold spots regions, the legitimate reflex would be to genotype markers that are as uniformly distributed as possible.

The simulation study confirms the higher efficiency of SNPs in linkage analysis compared with usual maps of microsatellites: the peak *z *score is higher with all scenarios of SNPs that we tested than with microsatellites (Figure 2) and among the resolutions of SNPs, analysis of maps of 0.2 cM and 0.5 cM densities found the exact position of the simulated gene. With the other two SNP densities (1 cM and 2 cM), the peak was 2 cM or less away from the simulated position, yet with the same significance level as at this position itself (smaller than 10^-4^). Unlike with microsatellites, the one *z *score interval contains the real simulated gene position for all four tested SNP densities. Therefore, the four densities of SNPs give comparable results in this simulation.

## Conclusion

A range of SNP densities was shown to be more efficient in linkage studies than the usual 10-cM microsatellites panel. Densities of approximately 1 SNP per centimorgan or per 2 cM should be favored because they give a satisfying power, accuracy, and precision, while denser maps can be the most useful in fine-gene mapping exploiting linkage disequilibrium. Alternatively, developing new linkage methods that are adapted to very dense marker maps may allow better extraction of the information contained in the maps.

## Abbreviations

COGA: Collaborative Study on the Genetics of Alcoholism

GAW14: Genetic Analysis Workshop 14

GH: GENEHUNTER

GH+: GENEHUNTER+

LD: Linkage disequilibrium

NPL: Nonparametric linkage

SNP: Single-nucleotide polymorphism

## Authors' contributions

JN performed data handling and analyses and drafted the manuscript. HR performed MERLIN acquisition and installation and contributed in data handling. DG supervised the work, gave advices on the analyses to perform and contributed in the interpretation of the results. All the authors read and approved the final version of the manuscript.

## Figures and Tables

**Table 1:** Regions with peaks of *z *scores and their *p*-values (SNP data)

Region (cM)	Density of 0.2 cM		Density of 0.5 cM		Density of 1 cM		Density of 2 cM	
	*z *score	*p*-value	*z *score	*p*-value	*z *score	*p*-value	*z *score	*p*-value
108–112	2.14	0.02	1.76	0.04	1.85	0.03	-	-
114–118	1.99	0.02	1.05	NS	-	-	1.93	0.03
119–121	1.82	0.03	1.43	NS	1.69	0.05	-	-
123–124	1.67	0.05	-	-	-	-	-	-
128–129	1.52	NS	1.34	NS	1.54	NS	-	-
135–138	1.72	0.04	1.51	NS	1.66	0.05	1.78	0.04
145–148	1.76	0.04	1.61	NS	-	-	-	-
